# Arterial Input Function (AIF) Correction Using AIF Plus Tissue Inputs with a Bi-LSTM Network

**DOI:** 10.3390/tomography10050051

**Published:** 2024-04-30

**Authors:** Qi Huang, Johnathan Le, Sarang Joshi, Jason Mendes, Ganesh Adluru, Edward DiBella

**Affiliations:** 1 Utah Center for Advanced Imaging Research (UCAIR), Department of Radiology and Imaging Sciences, University of Utah, Salt Lake City, UT 84108, USA; qi.huang@utah.edu (Q.H.); john.le@utah.edu (J.L.); jason.mendes@hsc.utah.edu (J.M.); ganeshsharma.adluru@hsc.utah.edu (G.A.); 2 Department of Biomedical Engineering, University of Utah, Salt Lake City, UT 84112, USA; sarang.joshi@utah.edu

**Keywords:** arterial input function, AIF correction, AIF saturation, deep learning, Bi-LSTM, myocardial perfusion MRI

## Abstract

**Background:** The arterial input function (AIF) is vital for myocardial blood flow quantification in cardiac MRI to indicate the input time–concentration curve of a contrast agent. Inaccurate AIFs can significantly affect perfusion quantification. **Purpose:** When only saturated and biased AIFs are measured, this work investigates multiple ways of leveraging tissue curve information, including using AIF + tissue curves as inputs and optimizing the loss function for deep neural network training. **Methods**: Simulated data were generated using a 12-parameter AIF mathematical model for the AIF. Tissue curves were created from true AIFs combined with compartment-model parameters from a random distribution. Using Bloch simulations, a dictionary was constructed for a saturation-recovery 3D radial stack-of-stars sequence, accounting for deviations such as flip angle, T2* effects, and residual longitudinal magnetization after the saturation. A preliminary simulation study established the optimal tissue curve number using a bidirectional long short-term memory (Bi-LSTM) network with just AIF loss. Further optimization of the loss function involves comparing just AIF loss, AIF with compartment-model-based parameter loss, and AIF with compartment-model tissue loss. The optimized network was examined with both simulation and hybrid data, which included in vivo 3D stack-of-star datasets for testing. The AIF peak value accuracy and $k^{trans}$ results were assessed. **Results**: Increasing the number of tissue curves can be beneficial when added tissue curves can provide extra information. Using just the AIF loss outperforms the other two proposed losses, including adding either a compartment-model-based tissue loss or a compartment-model parameter loss to the AIF loss. With the simulated data, the Bi-LSTM network reduced the AIF peak error from −23.6 ± 24.4% of the AIF using the dictionary method to 0.2 ± 7.2% (AIF input only) and 0.3 ± 2.5% (AIF + ten tissue curve inputs) of the network AIF. The corresponding $k^{trans}$ error was reduced from −13.5 ± 8.8% to −0.6 ± 6.6% and 0.3 ± 2.1%. With the hybrid data (simulated data for training; in vivo data for testing), the AIF peak error was 15.0 ± 5.3% and the corresponding $k^{trans}$ error was 20.7 ± 11.6% for the AIF using the dictionary method. The hybrid data revealed that using the AIF + tissue inputs reduced errors, with peak error (1.3 ± 11.1%) and $k^{trans}$ error (−2.4 ± 6.7%). **Conclusions**: Integrating tissue curves with AIF curves into network inputs improves the precision of AI-driven AIF corrections. This result was seen both with simulated data and with applying the network trained only on simulated data to a limited in vivo test dataset.

## 1. Introduction

Myocardial perfusion is significant in the evaluation of cardiovascular health, particularly in the context of ischemic heart disease, a condition characterized by reduced blood supply to the heart muscle [1]. Cardiac perfusion MRI, a non-invasive imaging modality, has emerged as a powerful tool for the quantification of myocardial blood flow (MBF) [2]. This technique involves the use of a gadolinium-based contrast agent (Gd), which is injected into the patient’s bloodstream. The passage of this paramagnetic agent through the myocardium is then tracked using MRI, allowing for the calculation of MBF. A quantitative approach provides a more precise assessment of myocardial perfusion, enabling clinicians to accurately diagnose ischemic heart disease, monitor disease progression, and evaluate the effectiveness of therapeutic interventions [3,4].

An accurate arterial input function (AIF) is a fundamental component in the quantification of myocardial blood flow using cardiac MRI [5,6,7]. The AIF represents the time–concentration curve of the contrast agent in the blood, serving as a reference for tracking the agent’s passage through the heart muscle. The AIF enables the calculation of key perfusion parameters such as myocardial blood flow and volume, which are instrumental in diagnosing and managing cardiovascular diseases. Given its central role, the accuracy of the AIF is critical. Inaccuracies in the AIF can lead to significant errors in perfusion quantification, potentially resulting in misdiagnoses or inappropriate treatment decisions [8].

The relationship between the MRI signal and Gd concentration is nonlinear, leading to signal saturation at high Gd concentrations. This saturation effect distorts the AIF, resulting in an underestimation of the peak contrast agent concentration. To correct the AIF for signal saturation, dual-bolus [9] and dual-sequence [10] techniques aim to minimize signal saturation by acquiring images after injecting a small pre-bolus of contrast agent or by using shorter saturation-recovery times (SRT), respectively. Prior studies have shown the feasibility of obtaining a short-SRT AIF with no added time cost [7,11]. Other approaches, such as blind estimation methods, have been used to obtain an accurate AIF without additional image acquisitions or contrast injections [12,13]. Most recently, machine learning algorithms have been employed to learn the nonlinear relationship between saturated and unsaturated signal intensity (SI) curves using a large dataset of images from over 200 patients [14]. This learned network displayed the corrected AIFs and demonstrated that the myocardial blood flow results calculated with the corrected AIFs were comparable to the reference values. However, the effectiveness of arterial input function correction diminishes notably when applied to infrequent cases that lie outside the bulk of the training data. To enhance the accuracy of the machine learning AIF approach, this study investigates including myocardial tissue signals obtained during the dynamic MRI acquisition.

## 2. Methods

### 2.1. Overview

The overall approach was to generate simulated data for training and testing in a Bi-LSTM neural network (NN) and compare the NN-corrected AIFs with the dictionary-based AIFs (inputs to the networks). Additionally, the training was adapted to apply the networks to 12 in vivo datasets.

Grammarly and ChatGPT-4 were used to aid in checking grammar and improving manuscript wording.

### 2.2. Data Preparation

#### 2.2.1. Simulated True AIF and Tissue Curves

The generation of simulated data employed a mathematical model for the true, unsaturated AIF [12]. The AIF model was a sum of three gamma variates and one sigmoid function, which were previously applied in the blind estimation of AIF [13]. The equation was represented in the following form:

(1) $$C_{AIF}\left(t\right)=\sum_{k=1,2,3}A_{k}G(\lambda_{k},\tau_{k},\Delta_{k})+A_{4}S({\lambda_{4},\tau }_{4},\Delta_{4},T)$$

where *G* represents a gamma variate, *S* is a sigmoid function, $A_{k}$ are scaling constants, $\Delta_{k}$ are delay time terms, $\lambda_{k}$ and $\tau_{k}$ are related to the shape and width of the first-pass bolus and recirculation peaks, and T represents the exponential time constant for contrast elimination from the blood pool. To reduce the number of parameters, Equation (1) was modified as below [13]:

(2) $$C_{AIF}=A_{1}G\left(\lambda_{1},\tau_{1},\Delta_{1}\right)+A_{2}G\left(\lambda_{2},\tau_{2},\Delta_{1}+\Delta_{2}\right)+A_{3}G\left(\lambda_{2},\tau_{2},\Delta_{1}+\Delta_{3}\right)+A_{4}S({\lambda_{2},\tau }_{3},\Delta_{1}+\Delta_{3},T)$$

Here, the parameter values were empirically chosen to approximate the shape of the real AIF curve population. To construct a realistic pool of AIF curves, we used $A_{1}\in \left[3,9\right]$, $\lambda_{1}\in \left[5,13\right]$, $\tau_{1}\in \left[0.015,0.025\right]$ min, $\Delta_{1}\in \left[0.1,0.15\right]$ min, $A_{2}\in \left[0.75,1.25\right]$, $\lambda_{2}\in \left[5,7\right]$, $\tau_{2}\in \left[0.15,0.25\right]$ min, $\Delta_{2}\in \left[0.04,0.05\right]$ min, $A_{3}\in \left[0.35,0.55\right]$, $\Delta_{3}\in \left[0.56,0.76\right]$ min, $A_{4}\in \left[2.5,3.5\right]$, and $T\in \left[0.35,0.55\right]$ min. A time interval of 0.5 s and a total of 120 time points were used for each AIF and the other simulated curves presented.

A four-parameter compartment model was used [15], described as follows:

(3) $$C_{tiss}(t)=C_{AIF}\left(t-\Delta t\right)⨂k^{trans}e^{-k_{ep}t}+v_{p}C_{AIF}\left(t-\Delta t\right)$$

where $k^{trans}$ is proportional to the myocardial blood flow (MBF), $k_{ep}$ is the parameter that controls the shape of tissue curves, $v_{p}$ is the portion of vasculature within the tissue, and Δt represents the enhancement time delay between the left ventricle blood pool and the myocardium. The four pharmacokinetic parameters were assigned values according to a realistic range reported in [15]. Specifically, $k^{trans}\in [0.3,1.1]$, $k_{ep}\in [1,5]$, $v_{p}\in [0,0.05]$, and $\Delta t\in \left[0,0.05\right]$ minute. Tissue curves were generated from the true AIFs described in Equation (2), with pharmacokinetic parameters sourced from a uniform random distribution.

#### 2.2.2. Saturated AIF

Bloch simulations were employed to construct a dictionary for a saturation-recovery 3D radial stack-of-star (SoS) sequence [16] to map between SI and Gd concentration. The noise-free true AIF Gd curves were converted to SI with the dictionary using sequence parameter settings from in vivo data from a retrospective study at 3T [7]. The in vivo data had a flip angle (FA) of 12° for the Gd-enhanced signal, 24 rays for a k-space center partition, SRT = 100 ms, TR = 2 ms, and TE = 1ms, native blood T1 of 1.8 s, and the non-contrast T2* value was assumed to be 0.06 s based on the measurement of average T2* of blood in a clinical myocardial T2* map. T1 and T2* relaxivity used 3.8 and 5.7 L/mmol-s [17].

The main sources causing bias of SI in AIF curves include the nonlinear signal response inherent to saturation recovery, T2* decay caused by high contrast agent concentration, imperfect saturation of magnetization post-SR pulse, FA bias due to B1 inhomogeneity, and spatial signal variations caused by sensitivity profiles of the surface coils [10]. The nonlinearity between SI and gadolinium concentration can be modeled [18]. Additionally, three factors—FA, T2*, and residual magnetization—were included in the simulation. The variation in coil sensitivities can be corrected using proton density images, and thus was not considered in the simulation.

Specifically, FA was altered by up to ±10%, while T2* and initial magnetization changed by a maximum of 10%. Training, validation, and test data all adhered to these ranges. The biased AIF SI curves were subsequently converted back to [Gd] curves using the nominal values of sequence parameters and the same dictionary. Gaussian noise was introduced to the AIF signal intensity (SI) curves rather than the [Gd] curves. This procedure was similarly applied to tissue curves to incorporate noise. The noise standard deviation was 5% of the peak value of tissue curves, roughly aligning with the pixel-wise noise value observed in the 12 in vivo datasets. The identical noise level was imposed on the saturated AIF SI curves.

### 2.3. Deep Neural Networks (DNNs)

Three different loss functions were implemented and compared using a Bi-LSTM network, including just AIF loss (Equation (4)), AIF with compartment-model parameter loss (Equation (5)), and AIF with compartment-model-based tissue loss (Equation (6)).

#### 2.3.1. Loss Functions

In Equation (4), an L1 loss is applied to the AIF loss, where $C_{AIF}$ represents the true (simulation data) or measured (in vivo data) Gd concentration time curves, and the hat symbol means predicted curves. The predicted curves, $\hat{C_{AIF}}$, with a time length of 120 points in this study were iteratively updated to minimize the loss function. In Equation (5), $\hat{Para}$ = {$\hat{k^{trans}}$, $\hat{k_{ep}}$, $\hat{v_{p}}$, $\hat{\Delta t}$}, *α* and *β* are the weights of each loss term, and *N* is the number of tissue curves. For in vivo data, true model parameters were calculated based on the target *AIF* and measured tissue curves. In Equation (6), *α* and δ are the weights of each loss term. The compartment-model parameters $\hat{Para}$ were combined with $\hat{C_{AIF}}$ to model the tissue loss, and $C_{tiss,n}$ represents true (simulation data) or measured (in vivo data) Gd concentration time curves.

(4) $$L1=\arg\underset{\hat{C_{\mathrm{AIF}}}}{\min}{\left\|C_{AIF}-\hat{C_{AIF}}\right\|}_{1}$$

(5) $$L2=\arg\underset{\hat{C_{\mathrm{AIF}}},\hat{\mathrm{Para}}}{\min}\alpha {\left\|C_{AIF}-\hat{C_{AIF}}\right\|}_{1}+\beta \frac{1}{N}\sum_{n=1}^{N}{\left\|Para-\hat{Para}\right\|}_{1}$$

(6) $$L3=\arg\underset{\hat{C_{\mathrm{AIF}}},\hat{\mathrm{Para}}}{\min}\alpha {\left\|C_{AIF}-\hat{C_{AIF}}\right\|}_{1}+\delta \frac{1}{N}\sum_{n=1}^{N}{\left\|C_{tiss,n}-\hat{C_{AIF}}\left(t-\Delta t\right)⨂\hat{k^{trans}}e^{-\hat{k_{ep}}t}+\hat{v_{p}}\hat{C_{AIF}}\left(t-\Delta t\right)\right\|}_{1}$$

Initially, two important factors needed to be optimized for the AIF correction, including the number of tissue curves (or inputs to the network) and the selection of the loss function. In the analysis to determine the optimal number of tissue curves, the $k^{trans}$ values were set to range from 0.3 to 2.3, increasing by intervals of 0.2 for each additional tissue curve. Consequently, the surveyed number of curves varied between 0 (just AIF input) and 10. Moreover, $k_{ep}$ will change accordingly as the extravascular extracellular space is restricted within 0.2–0.3 [19,20]. The second experiment was investigated based on the comparison of the loss functions described in Equations (4)–(6).

#### 2.3.2. Networks

##### Bidirectional Long Short-Term Memory (Bi-LSTM)

**Figure 1:**
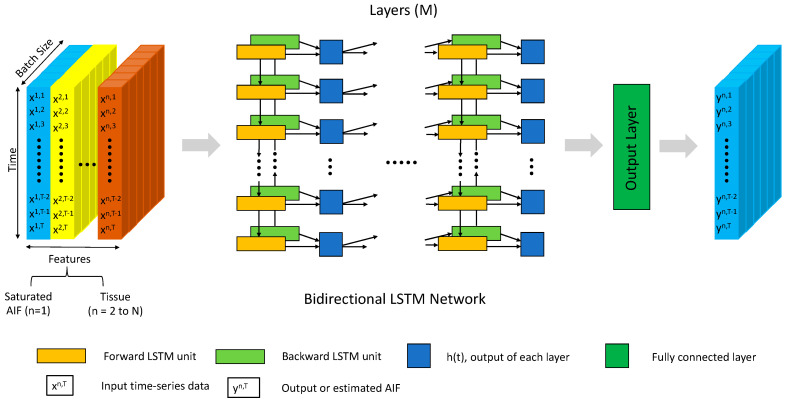
The workflow of AIF corrections using a Bi-LSTM network. The input is an incorrect AIF with tissue curves, and the output is a network-predicted AIF. The main body of the network uses a Bi-LSTM architecture consisting of multiple layers (M = 4), each incorporating both a forward and a backward LSTM unit to process time-series data.

A 1D Bi-LSTM network [21] was first implemented to evaluate its efficacy in AIF corrections by studying the three factors to find the optimal setting (decision on the format of the loss function and inputs to networks) for AIF corrections. The workflow for AIF corrections with the Bi-LSTM network is displayed in Figure 1. The Bi-LSTM network consisted of four layers, each including a forward and backward LSTM unit. The number of hidden nodes inside each LSTM unit was 32. After the concatenation of outputs from the last layer of Bi-LSTM, a linear layer was employed to convert feature size to the time length of AIF as the predicted AIF output.

The network was implemented based on the Pytorch platform. The inputs to the network were the inaccurate AIFs generated with the dictionary method. These same AIFs were used as a baseline to indicate improvements in AIF estimates.

##### Training, validation, and test datasets

In the following studies with simulated data, 10,000 sets of time curves were generated randomly. Of the total sets, 8000 sets were used for training, 1000 sets for validation, and 1000 sets for testing. Each set included the saturated and biased AIFs along with the true unsaturated AIF curves, alongside up to 10 tissue curves. The unsaturated AIFs were noise-free true AIF curves derived from the mathematical model, as seen in Equation (2).

#### 2.3.3. Hyperparameters

The networks used a batch size of 16, 100 training epochs, and ADAM optimization with a learning rate of 0.0003. All network parameters were initialized as zero for “bias” and He normal weights [22] for “weight”. The best network was saved with the highest validation accuracy, while all loss curves were observed for a sanity check to avoid overfitting.

#### 2.3.4. Evaluation Metrics

Two metrics were applied to assess the accuracy of the predicted AIFs. Since signal saturation was most prominent at the peak concentration of an AIF, peak values between the estimated AIF and the reference were compared using percentage error. In addition, predicted AIFs and tissue curves were fitted using the Levenberg–Marquardt algorithm [23] to obtain the resulting pharmacokinetic parameters. The value of $k^{trans}$ was then used as the second metric to compare with the target because it is proportional to myocardial blood flow. The percentage error of the two indexes can be calculated using the equation below.

(7) $$Err\%=\frac{P-T}{T}$$

where *P* represents the predicted AIF peak or $k^{trans}$ values, and *T* indicates the target values. The error is visualized with an error bar plot.

Statistical analysis was conducted to assist in the visualization of results. A straight line produced from the linear fit can be drawn using the estimated slope and intersect values displayed in the scatter plot of AIF peak values, or $k^{trans}$, from the test data. Pearson coefficients were calculated to indicate the correlations between estimates and target values, and Bland–Altman plots were used to assess accuracy and outliers.

### 2.4. Applying the Trained Networks to In Vivo Data

Existing in vivo 3D radial SoS datasets were processed to determine how the simulation-trained networks performed on in vivo data. The in vivo acquisition includes a 2D AIF with SRT = 20 ms and 3D myocardial perfusion images with SRT = 100 ms. Other data acquisition details match the previous descriptions of generating the simulated data.

A region of interest in the left ventricle blood pool in the central slice from the long SRT 3D scan in five dogs (rest and stress scans) and two human subjects (rest) was employed to generate “saturated AIFs”. Subsequently, a preprocessing step, encompassing interpolation and alignment, was undertaken to ensure data format uniformity. The interpolation was set to a time interval of 0.5 ms and a total duration of 1 min, ensuring a consistent input length for the neural network. Furthermore, aligning AIFs from different subjects was essential due to potential variations in their timestamps.

Poor results were obtained from the trained networks applied to in vivo data. Thus, a new training set with FA altered one-sided by up to 10% was created, while a validation set was built with a bias range of up to 15% in order to account for the difference in data distribution between simulated and in vivo data. The same variations were applied to T2* and initial magnetization to mix the bias factors together.

For the hybrid dataset, both added noise and noise-free simulated curves were used for training. Noise-free simulated data were used for the final report of results because the noise-free simulated datasets gave superior results over noisy datasets. No in vivo data were used in the training set.

The hybrid datasets consisted of 8000/1000 sets of simulated time curves for training/validation and 12 sets of in vivo time curves for testing. Four tissue curves were generated with simulations for each AIF set for training and validation. The same number of tissue curves (four) were generated using K-means clustering from pixel-wise time curves within the myocardium for testing only.

## 3. Results

We used the Bi-LSTM network with simulated data to determine the optimal number of tissue curves (Section 3.1) and the choice of loss functions (Section 3.2). Section 3.3 gives results from the hybrid dataset described in Section 2.4 applied to the network with the same hyperparameter settings.

### 3.1. The Number of Tissue Curves

**Figure 2:**
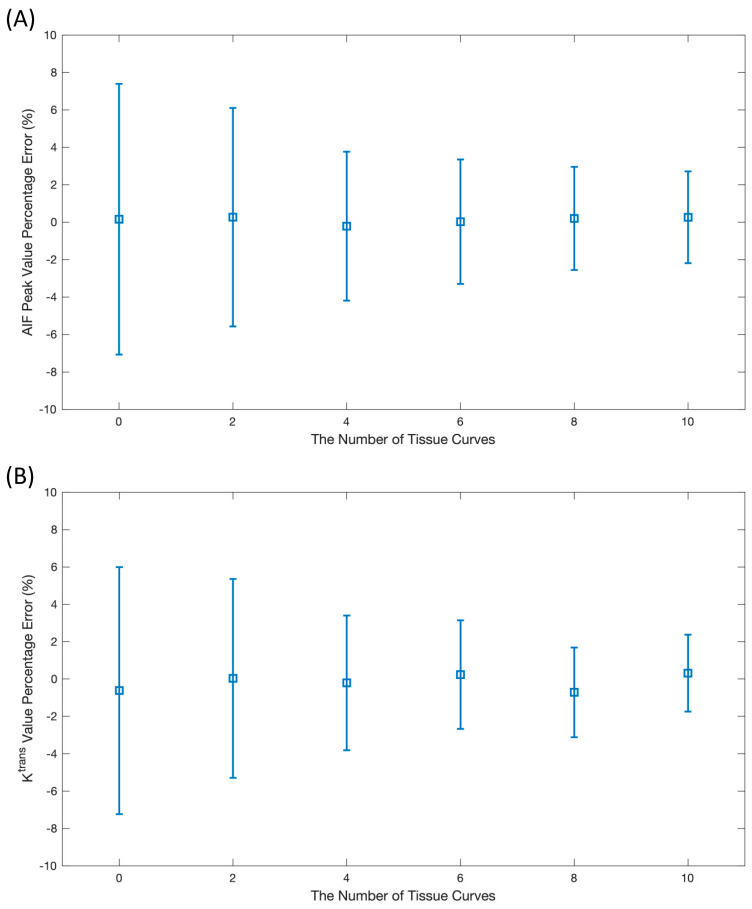
The investigation of the optimal number of tissue curves for the network input. The case of no tissue indicates the application of the AIF-only loss, while the number of tissue curves varied from 1 to 10. The AIF peak value error in (**A**) and the $k^{trans}$ error in (**B**) were two evaluation metrics.

Figure 2 demonstrates how the number of tissue curves, which extends from 0 (solely AIF input) to 10, influences AIF corrections. A substantial decrease in AIF peak error was observed with an increasing number of tissue curves, dropping from 0.2 ± 7.2% to 0.3 ± 2.5%. This pattern was mirrored in the associated $k^{trans}$ values, improving from −0.6 ± 6.6% to 0.3 ± 2.1%. Consequently, ten tissue curves were adopted for use in the subsequent simulation data studies.

### 3.2. The Comparison of Three Loss Functions

Table 1 presents a comparison of the effects of three loss functions on the AIF peak and the derived $k^{trans}$ values. When the weight ratio (either α:β or α:δ) was equal to 1:0, the network received only the saturated AIF curves as input. The table reveals no notable change in the percentage error of the AIF peak and $k^{trans}$ with an increase in β, whereas a significant increase in error was evident with a higher δ value.

**Figure 3:**
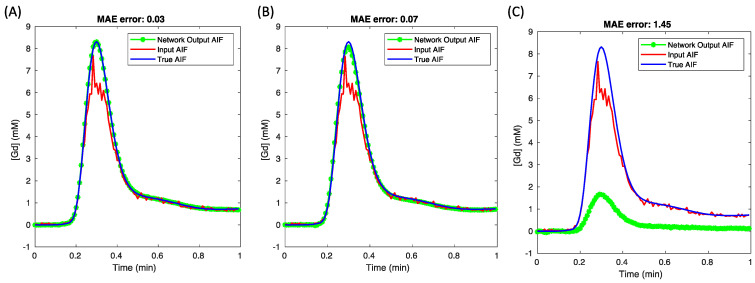
The comparison of network-predicted AIFs produced with the three proposed loss functions. The most accurate result was achieved using only the AIF loss, as shown in (**A**), with a mean absolute error (MAE) of 0.03. A slightly higher MAE was observed with the addition of parameter loss, as indicated in (**B**), while the AIF plus tissue loss, presented in (**C**), led to a significant underestimation in the network’s AIF prediction.

Figure 3 offers a visual comparison of AIF curves, including the input, the network output, and the true (target) AIF curves. Three cases using distinct loss functions are shown, where both AIF with parameter loss and AIF with model-based tissue loss were visualized using a weight ratio of 1:100, in contrast to just AIF loss. The findings suggest that using just AIF loss (with AIF + tissue inputs) was adequate for AIF corrections in this simulation dataset.

### 3.3. Comparison of AIF Inputs Only and AIF + Tissue Inputs: Hybrid Dataset

**Figure 4:**
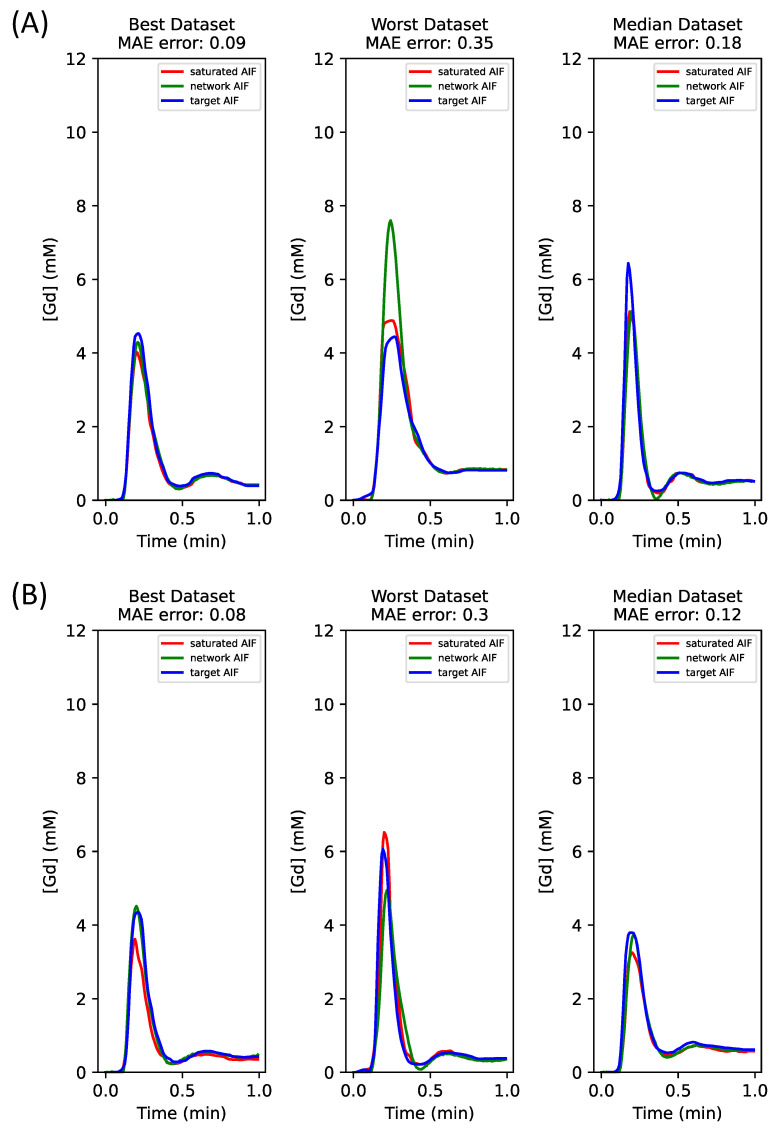
The comparison of AIF curves when trained exclusively with AIF-only input (**A**) or in combination with tissue curves (**B**), using the hybrid data. The input AIF curve, generated using the dictionary method (in green), serves as a baseline to highlight improvements in the network-predicted AIF. The AIF plus tissue inputs yielded a lower MAE across the board—best, median, and worst—compared to the AIF-only input.

In this study utilizing a hybrid dataset (with simulated training and in vivo data testing), significant improvements in the network-predicted values were observed after incorporating tissue curves into the input (as shown in Figure 4). Figure 4 labels the input AIF as “saturated AIF”—this is the AIF estimated by the Bloch equations dictionary processing of the saturated blood pool on the long SRT images. For this initial AIF input to the network (depicted in red in Figure 4A), the peak value percentage error was 10.4 ± 12.4%. Training the network with only the AIF input under the AIF loss resulted in error rates of 3.5 ± 25.6% for the Bi-LSTM-predicted AIF. However, by integrating AIF + tissue inputs with the AIF loss, the error rates improved to 1.3 ± 11.1% for the Bi-LSTM-predicted AIF. A similar enhancement is evident in Figure 4B, where the $k^{trans}$ percentage error decreased from −17.0 ± 16.6% for the initial input AIF (red in Figure 4B) to −10.4 ± 15.9% using just AIF input and to −2.4 ± 6.7% with the combined AIF + tissue inputs.

**Figure 5:**
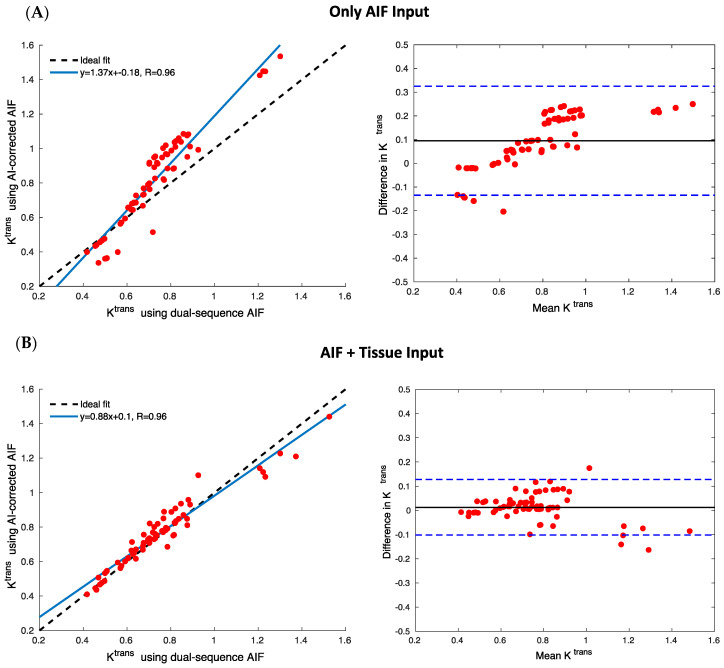
Statistical analysis of $k^{trans}$ values for two different network inputs (AIF-only and AIF + tissue) using the hybrid data. The Bland–Altman plot illustrates the difference between $k^{trans}$ values derived from network AIFs and true AIFs from the test set. The correlation plot showcases the linear fit (with a blue line), while the black dotted line represents the ideal fit. Each red circle above represents $k^{trans}$ from a slice.

Figure 5 shows the enhanced-accuracy $k^{trans}$ values estimated with the network AIFs, transitioning from the AIF-only input (Figure 5A) to the AIF + tissue inputs (Figure 5B). The linear fit for Figure 5A is y = 1.37x + 0.18 with a Pearson coefficient (R) of 0.96, whereas Figure 5B shows y = 0.88x + 0.10 with a R of 0.96. Despite similar R values, Figure 5B displays a closer match to the ideal fit line (dotted black line). Band–Altman analysis indicates a substantial reduction in mean bias (from 0.10 in AIF-only input to 0.01 in AIF + tissue inputs) and narrower 95% confidence intervals (from [−0.13, 0.33] for AIF-only input to [−0.10, 0.13] in AIF + tissue inputs), comparing network AIFs with the target AIFs obtained with the dual-sequence method.

## 4. Discussion

In this work, we have established that combining the AIF and tissue inputs under the AIF loss enhances the deep learning correction of signal bias in the AIF for quantitative perfusion CMR. The optimization began with a fundamental network architecture that used the AIF input with the AIF loss. The addition of tissue curves to the training inputs yielded improved AI correction. While increasing the number of tissue curves for network inputs refined the estimation, incorporating additional loss terms, such as compartment-model-based parameter loss and tissue loss, did not prove advantageous. Therefore, we focused on AIF + tissue curve inputs with the AIF loss, utilizing both purely simulated and hybrid datasets. This consistently demonstrated enhanced accuracy following the inclusion of tissue curves in the network inputs.

**Figure 6:**
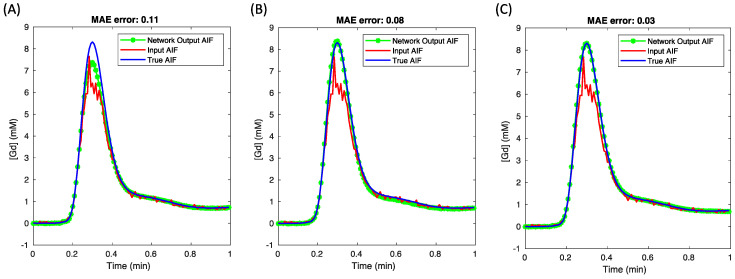
The comparison of network-predicted AIFs produced with the three ways of network inputs. Across scenarios (**A**–**C**), where the network was fed with AIF-only, tissue-only, and AIF plus tissue inputs, respectively, the AIF plus tissue inputs resulted in the most precise AIF estimates, achieving the lowest MAE in comparison to the other input methods.

Integrating tissue curves as network inputs bolsters the accuracy and precision of AIF predictions. This is because tissue curves embody a lower gadolinium concentration range, within which the relationships between gadolinium concentration and signal intensity remain linear, thus ensuring a linear correlation with the accurate AIF. Conversely, the saturated AIF exhibits a nonlinear relationship with the true AIF at higher concentrations. We validated this hypothesis by evaluating various network inputs, including just AIF-only, tissue-only, and AIF + tissue curves (Figure 6). The network-predicted AIF using AIF-only input (Figure 6A) displayed a distinctly saturated peak, whereas using only tissue curve inputs (Figure 6B) mitigated the peak value error. Given that the lower-concentration segment of the AIF input is expected to be unbiased, combining AIF and tissue curves as inputs (Figure 6C) achieves the most precise AIF estimates.

The finding that adding tissue curves may benefit accurate AIF estimation depends on the number of tissue curves and the diversity of the tissue curves. In the simulation study, a strategy that used an increment of 0.2 for given $k^{trans}$ values of multiple tissue curves was adopted empirically. Since increasing the number of tissue curves adds fresh information to inputs, using ten tissue curves can continue to improve AIF correction, as shown in Figure 2. However, this was not true for in vivo data. Healthy subjects tend to have relatively similar myocardial tissue curves; thus, only a small number of tissue curves clustered from a group of pixel-wise tissue curves may be sufficient. In this work, four tissue curves were found to enable the best performance for the in vivo data (not shown). Furthermore, patients with focal perfusion defects may have more diverse tissue clusters, thus requiring more tissue curves for inputs but offering possible performance improvements from more diversity.

Generating a simulation dataset is useful for studying deep learning for the AIF correction task, especially when there has been a lack of clinical sequences for quantitative myocardial perfusion. Without the assessment of open datasets and the lack of multi-center cooperation, the number of patient datasets to experiment with is limited. The simulation study is feasible for this work because the mathematical AIF model for generating AIF time curves and a compartment model for producing tissue time curves have been applied in previous works on AIF corrections [13]. Due to the similarity in shape between a gamma function and AIF, a gamma variate has been modified to model either the first-pass perfusion [24,25] or the whole perfusion process [12,26]. The model used for the first-pass perfusion requires fewer unknown parameters and can be combined with a Fermi model for quantitative analysis. However, this work adopted a 12-parameter AIF model to flexibly adjust simulated AIF curves to approximate real AIF curves.

Traditional machine learning operates on the premise that training and test sets hail from identical distributions. However, this may not always be the case in real-world scenarios, especially when datasets originate from disparate sources, multiple imaging centers, or become outdated due to evolving data over time. In this context, our approach used the assumption that in vivo test data presented a more diverse data distribution compared to simulated data [27].

Although the results section details the selection of loss functions, the rationale for why additional loss terms beyond AIF loss did not yield benefits remains unexplained. The premise behind the parameter loss was that these parameters operate on a different intensity scale compared to the values of the AIF curves. Furthermore, the tissue loss, derived from the compartment model, entailed convolving the estimated parameters with an estimated AIF, resulting in non-unique outcomes. This was demonstrated in Figure 4, where the network AIF estimate was obviously biased when using a combination of AIF and model-based tissue loss, applying a ratio of 1:100 for the two loss terms.

In a broader context, the results of this study have implications for the fields of cardiovascular imaging and patient care. If quantitative myocardial perfusion measurements can be obtained from MRI methods that do not need the acquisition or analysis of a separate dual bolus or dual sequence method, this simplifies the approach and can aid in its adoption. More widespread use of quantitative myocardial perfusion promises a more accurate diagnosis of ischemic heart diseases.

While the present study shows promising results for AIF correction, certain limitations may exist. The mathematical model for the AIF was previously validated to achieve good agreement between the blind estimated AIF and the measured AIF [12]. The mean bias and uncertainties of compartment-model parameters derived from the two AIFs were found to be comparable. For example, the mean bias of $k^{trans}$ was +7% and the uncertainty was 0.0043 min^−1^ for normal brain tissue. In addition, the simulated tissue curves were drawn from a uniform distribution, which reflects a mix of normal and abnormal perfusion and washout. The diversity of *k_ep_* is known from blind estimation studies to provide more information regarding the AIF [13]. As well, the use of simulated datasets, while advantageous for controlled experimentation, does not replicate real-world scenarios. Therefore, there is a need to validate these findings with larger, diverse, and real-world datasets to understand the broader applicability of the results. The selection of a Bi-LSTM network is due to its outstanding performance in handling time-series data; however, other advanced networks, such as transformers and gated recurrent units, may be better choices for AIF corrections. Therefore, future development of new networks is important, especially for tackling hybrid data better.

## 5. Conclusions

This work compares different ways of leveraging tissue curve data and determines that training a network using AIF + tissue curve inputs with an AIF loss alone is an effective approach to correct biased AIFs for quantitative myocardial perfusion MRI studies. The improvement of network-predicted AIFs using the proposed method is significant compared to the baseline results and to the use of only the AIF input, especially when the network training used simulated data and the testing used in vivo data. The combined AIF and tissue curve inputs improve the accuracy and precision of network-predicted AIF and make an AI-AIF approach more robust to outliers, which may simplify scanning and processing.

## Figures and Tables

**Table 1:** The percentage error of AIF peak value and $k^{trans}$ produced using the three loss functions applied with various weight ratios of loss terms.

Weights Ratio	AIF Peak Value Error %	*k^trans^* Error %
1:0 (or AIF loss only)	0.3 ± 2.5	0.3 ± 2.1
*α*:*β* = 1:1	0.9 ± 2.5	–1.0 ± 2.3
*α*:*β* = 1:10	0.2 ± 3.2	1.1 ± 3.4
*α*:*β* = 1:100	–1.3 ± 4.4	2.3 ± 4.1
*α*:*δ* = 1:1	0.7 ± 4.1	0.4 ± 3.3
*α*:*δ* = 1:10	76.5 ± 2.6	–337.4 ± 48.8
*α*:*δ* = 1:100	78.2 ± 2.3	–374.1 ± 43.0

## Data Availability

The data presented in this study are available on reasonable request from the corresponding author.
